# Managing variability in the summary and comparison of gait data

**DOI:** 10.1186/1743-0003-2-22

**Published:** 2005-07-29

**Authors:** Tom Chau, Scott Young, Sue Redekop

**Affiliations:** 1 Bloorview MacMillan Children's Centre, Toronto, Canada; 2 Institute of Biomaterials and Biomedical Engineering, University of Toronto, Toronto, Canada

## Abstract

Variability in quantitative gait data arises from many potential sources, including natural temporal dynamics of neuromotor control, pathologies of the neurological or musculoskeletal systems, the effects of aging, as well as variations in the external environment, assistive devices, instrumentation or data collection methodologies. In light of this variability, unidimensional, cycle-based gait variables such as stride period should be viewed as random variables and prototypical single-cycle kinematic or kinetic curves ought to be considered as random functions of time. Within this framework, we exemplify some practical solutions to a number of commonly encountered analytical challenges in dealing with gait variability. On the topic of univariate gait variables, robust estimation is proposed as a means of coping with contaminated gait data, and the summary of non-normally distributed gait data is demonstrated by way of empirical examples. On the summary of gait curves, we discuss methods to manage undesirable phase variation and non-robust spread estimates. To overcome the limitations of conventional comparisons among curve landmarks or parameters, we propose as a viable alternative, the combination of curve registration, robust estimation, and formal statistical testing of curves as coherent units. On the basis of these discussions, we provide heuristic guidelines for the summary of gait variables and the comparison of gait curves.

## Introduction

### Definition of variability

In quantitative gait analysis, variability is commonly understood to be the fluctuation in the value of a kinematic (e.g. joint angle), kinetic (e.g. ground reaction force), spatio-temporal (e.g. stride interval) or electromyographic measurement. This fluctuation may be observed in repeated measurements over time, across or within individuals or raters, or between different measurement, intervention or health conditions. In this paper, we will focus on the variability in two types of data: unidimensional gait variables and single-cycle, prototypical gait curves, as these are the most common abstractions of spatio-temporal, kinematic and kinetic data, typically collected within a gait laboratory.

### Measurement

Many different analytical methods have been proposed for estimating the variability in gait variables. The most widely used measures are those relating to the second moment of the underlying probability distribution of the gait variable of interest. Examples include, standard deviation (e.g., [1-4]), coefficient of variation (e.g., [5-8]) and coefficient of multiple correlation (e.g., [9,10]). Other less conventional variability measures have also been suggested. For example, Kurz et al. demonstrated an information-theoretic measure of variability, where increased uncertainty in joint range-of-motion (ROM), and hence entropy, reflected augmented variability in joint ROM [11].

For gauging variability among gait curves, some distance-based measures have been put forth, including the mean distance from all curves to the mean curve in raw 3-dimensional spatial data [12], the point-by-point intercurve ranges averaged across the gait cycle [13] and the norm of the difference between coordinate vectors representing upper and lower standard deviation curves in a vector space spanned by a polynomial basis [14]. Instead of reporting a single number, an alternative and popular approach to ascertain curve variability has been to peg prediction bands around a group of curves. Recent research on this topic has demonstrated that bootstrap-derived prediction bands provide higher coverage than conventional standard deviation bands [15-17].

Additionally, various summary statistics, such as the intra-class correlation coefficient [8] and Pearson correlation coefficient [18], for estimating gait measurement reliability, repeatability or reproducibility have been deployed in the assessment of methodological, environmental and instrumentation or device-induced variability. Principal components and multiple correspondence analyses have also been applied in the quantification of variability in both gait variables and curves, as retained variance and inertia, respectively, in low dimensional projections of the original data [19].

### Sources of variability

**Figure 1:**
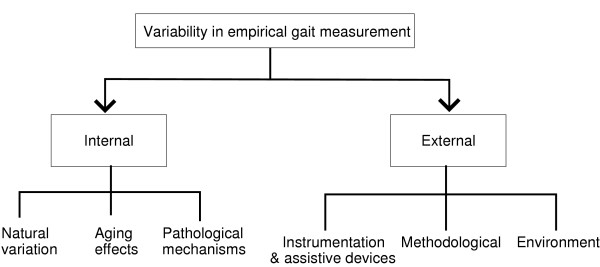
Sources of variability in empirical gait measurements.

As depicted in Figure 1, the numerous sources of variability in gait measurements can be loosely categorized as either internal or external to the individual being observed [20].

#### Internal

Internal variability is inherent to a person's neurological, metabolic and musculoskeletal health, and can be further subdivided into natural fluctuations, aging effects and pathological deviations. It is now well known that neurologically healthy gait exhibits natural temporal fluctuations that are governed by strong fractal dynamics [21-23]. The source of these temporal fluctuations may be supraspinal [24] and potentially the result of correlated central pattern generators [25]. One hierarchical synthesis hypothesis purports that these nonlinear dynamics are due to the neurological integration of visual and auditory stimuli, mechanoreception in the soles of the feet, along with vestibular, proprioceptive and kinesthetic (e.g., muscle spindle, Golgi tendon organ and joint afferent) inputs arriving at the brain on different time scales [24,26]. Internal variability in gait measurements may be altered in the presence of pathological conditions which affect natural bipedal ambulation. For example, muscle spasticity tends to augment within-subject variability of kinematic and time-distance parameters [10] while Parkinson's disease, particularly with freezing gait, leads to inflated stride-to-stride variability [27] and electromyographic (EMG) shape variability and reduced timing variability in the EMG of the gastrocnemius muscle [28]. Similarly, recent studies have reported increased stride-to-stride variability due to Huntington's disease [29], amplified swing time variability due to major depressive and bipolar disorders [30], and heightened step width [31] and stride period [32] variability due to natural aging of the locomotor system.

#### External

Aside from mechanisms internal to the individual, variability in gait measurements may also arise from various external factors, as shown in Figure 1. For example, influences of the physical environment, such as the type of walking surface [33], the level of ambient lighting in conjunction with type of surface [34] and the presence and inclination of stairs [35] have been shown to affect cadence, step-width, and ground reaction force variability, respectively, in certain groups of individuals. Assistive devices, such as canes or semirigid ankle orthoses may reduce step-time and step-width variability [36] while different footwear (soft or hard) can affect the variability of knee and ankle joint angles, possibly by altering peripheral sensory inputs [14].

Variability may also originate from the nature of the instrumentation employed. This variability is often appraised by way of test-retest reliability studies. Some recent examples include the reproducibility of measurements made with the GAITRite mat [8], 3-dimensional optical motion capture systems [9,18], triaxial accelerometers [37], insole pressure measurement systems [4], and a global positioning system for step length and frequency recordings [7].

Experimenter error or inconsistencies may also contribute, as an external source, to the observed variability in gait data. Besier et al. contend that the repeatability of kinematic and kinetic models depends on accurate location of anatomical landmarks [38]. Indeed, various studies have confirmed the exaggerated variability in kinematic data due to differences in marker placement between trials [9,39] and between raters [40]. Finally, analytical manipulations, such as the computation of Euler angles [9] or the estimation of cross-sectional averages [41] may also amplify the apparent variability in gait data.

### Clinical significance of variability

The magnitude of variability and its alteration bears significant clinical value, having been linked to the health of many biological systems. Particularly in human locomotion, the loss of natural fractal variability in stride dynamics has been demonstrated in advanced aging [32] and in the presence of neurological pathologies such as Parkinson's disease [42], and amyotrophic lateral sclerosis [42]. In some cases, this fractal variability is correlated to disease severity [32]. Variability may also serve as a useful indicator of the risk of falls [43] and the ability to adapt to changing conditions while walking [44]. Stride-to-stride temporal variability may be useful in studying the developmental stride dynamics in children [45]. Natural variability has been implicated as a protective mechanism against repetitive impact forces during running [14] and possibly a key ingredient for energy efficient and stable gait [46]. Variability is not always informative and useful and in fact may lead to discrepancies in treatment recommendations. For example, due to variability in static range-of-motion and kinematic measurements, Noonan et al. found that different treatments were recommended for 9 out of 11 patients with cerebral palsy, examined at four different medical centres [13].

### Dealing with variability

Given the ubiquity and health relevance of variability in gait measurements, it is critical that we summarize and compare gait data in a way that reflects the true nature of their variability. Despite the apparent simplicity of these tasks, if not conducted prudently, the derived results may be misleading, as we will exemplify. In fact, there are to date many open questions relating to the analysis of quantitative gait data, such as the elusive problem of systematically comparing two families of curves.

The objectives of this paper are twofold. First, we aim to review some of the analytical issues commonly encountered in the summary and comparison of gait data variables and curves, as a result of variability. Our second goal is to demonstrate some practical solutions to the selected challenges, using real empirical data. These solutions largely draw upon successful methods reported in the statistics literature. The remainder of the paper addresses these objectives under two major headings, one on gait variables and the other on gait curves. The paper closes with some suggestions for the summary and comparison of gait data and directions for future research on this topic.

## Gait random variables

Unidimensional variables which are measured or computed once per gait cycle will be referred to as gait random variables. This category includes spatio-temporal parameters such as stride length, period and frequency, velocity, single and double support times, and step width and length, as well as parameters such as range-of-motion of a particular joint, peak values, and time of occurrence of a peak, which are extracted from kinematic or kinetic curves on a per cycle basis.

Due to variability, univariate gait measures and parameters derived thereof should be regarded as stochastic rather than deterministic variables [47,48]. In this random variable framework, a one-dimensional gait variable is represented as **X **and governed by an underlying, unknown probability distribution function *F*_*X*_, or density function . A realization of this random variable is written in lower case as *x*.

### Inflated variability and non-robust estimation

It has been recently demonstrated that typical location and spread estimators used in quantitative gait data analysis, i.e. mean and variance, are highly susceptible to small quantities of contaminant data [48]. Indeed, a few spurious or atypical measurements can unduly inflate non-robust estimates of gait variability. The challenge in the summary of highly variable univariate gait data lies in reporting location and spread, faithful to the underlying data distribution and minimally influenced by extraordinary observations.

Here, we focus on the issue of inflated variability and non-robust estimation by examining four different spread estimators, applied to stride period data from a child with spastic diplegic cerebral palsy. As stated above, the coefficient of variation and standard deviation are routinely employed in the summary of gait variables. Given a sample of *N *observations of a gait variable **X**, i.e., {*x*_1_,..., *x*_*N*_}, the coefficient of variation is defined as,



where the numerator is simply the sample standard deviation and the denominator, , is the sample mean. We also include two other estimators, although seldom used in gait analysis, to illustrate the qualitative differences in estimator robustness. The interquartile range of the sample is defined as

**Figure 2:**
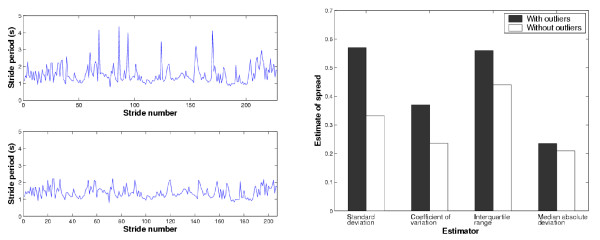
Robust vs. non-robust estimators of parameter spread. The left pane shows a sequence of stride periods with outliers (top) and after removal of outliers (bottom). The right pane is a bar graph showing the values of four different spread estimators before and after outlier removal.

*IQR*(**X**) = *x*_0.75 _- *x*_0.25 _    (2)

where *x*_0.75 _and *x*_0.25 _are the 75% and 25% quantiles. The *q*-quantile is defined as  where as usual, *F*_*X *_is the probability distribution of **X**. Equivalently, the *q*-quantile is the value, *x*_*q*_, of the random variable where . That is, *q *× 100 percent of the random variable values lie below *x*_*q*_. We also introduce the median absolute deviation [49],

*MAD*(**X**) = med (|**X **- med(**X**)|)     (3)

where med(*X*) is the median of the sample, or the 50% quantile as defined above. This last estimator is, as the name implies, the median of the absolute difference between the sample values and their median value. We are interested in studying how these different estimators perform when estimating the spread in a gait variable, the observations of which may contain outlying values or contaminants. In the left pane of Figure 2, we show a set of stride period data recorded from a child with spastic diplegia. The top graph shows the raw data with a number of obvious outliers with atypically long stride times. We adopted a common outlier definition, labeling points more than 1.5 interquartile ranges away from the sample median as extreme values. According to this definition there were 21 outlying observations. In the bottom graph, the outliers have been removed. The bar graph on the right-hand side of Figure 2 portrays the spread estimates of the stride period data, computed with each estimator introduced above, with and without the outliers.

We note immediately that the spread estimates in the presence of outliers are higher. The standard deviation and coefficient of variation change the most, dropping 42 and 36 percent in value, respectively, upon outlier removal. This observation is particularly important in the comparison of gait variables, as inflated variability estimates will diminish the probability of detecting significant differences when they do in fact exist. In contrast, the interquartile range and median absolute deviation, only change by 21 and 11%, respectively. We see that these latter estimates are more statistically stable, in that they are not as greatly influenced by the presence of extreme observations.

To more fully comprehend estimator robustness or lack thereof, the field of robust statistics offers a valuable tool called influence functions, which as the name implies, summarizes the influence of local contaminations on estimated values. Their use in gait analysis was first introduced in the context of stride frequency estimation [48].

We first introduce the concept of a functional, which can be understood as a real-valued function on a vector space of probability distributions [50]. In the present context, functionals allow us to think of an estimator as a function of a probability distribution. For example, for the interquartile range, the functional is simply, .

**Figure 3:**
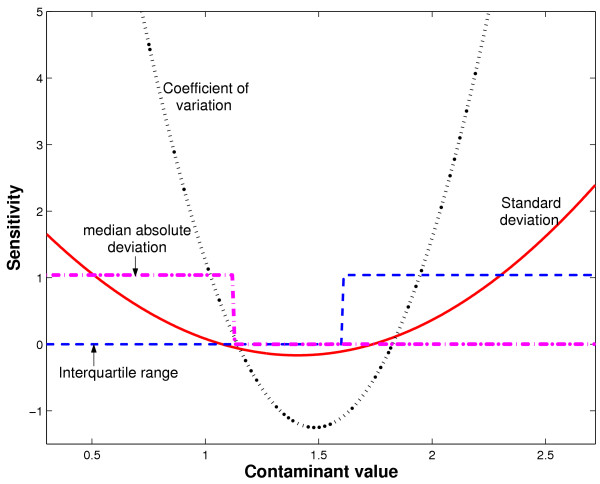
Sensitivity curves for various estimators of gait parameter variability based on the stride period example.

Let the mixture distribution *F*_*z*, *ε *_describe data governed by distribution *F *but contaminated by a sample *z*, with probability *ε*. The influence function at the contamination *z *is defined as



where *T*(·) is the functional for the estimator of interest. The influence function for a particular estimator measures the incremental change in the estimator, in the presence of large samples, due to a contamination at *z*. Clearly, if the impact of this contaminant on the estimated value is minimal, then the estimator is locally robust at *z*. Influence functions can be analytically derived for a variety of common gait estimators (see for example, [48]), including those mentioned above. For the sake of analytical simplicity and practical convenience, we will instead use finite sample sensitivity curves, *SC*(*z*), which can be defined as,

*SC*(*z*) = (*N *+ 1){*T*(*x*_1_,..., *x*_*N*_, *z*) - *T*(*x*_1_,..., *x*_*N*_)}     (5)

where as above, *T*(·) is the functional for the estimator in question, and *z *is the contaminant observation. When *N *→ ∞ the sensitivity curve converges to the influence function for many estimators. Like the asymptotic influence functions, sensitivity curves describe the local impact of a contamination *z *on the estimator value. For the purposes of computer simulation, the functional *T*(*x*_1_,..., *x*_*N*_, *z*) and *T*(*x*_1_,..., *x*_*N*_) are simply the evaluations of the estimator of interest at the augmented and original samples, respectively. Figure 3 depicts the sensitivity curves for the estimators introduced in the stride period example. To generate these curves, we used the cleansed stride period data (without outliers) and incrementally added a deviant stride period from 0.5 below the lowest sample value to 0.5 above the highest sample value. The sample mean for this data was 1.41 seconds.

We observe that both standard deviation and coefficient of variation have quadratic sensitivity curves with vertices close to the sample mean. In other words, as contaminants take on extreme low or high values, the estimated values are unbounded. Clearly, these two estimators are not robust, explaining their high sensitivity to the outliers in the stride period data. In contrast, both the interquartile range and median absolute deviation have bounded sensitivity curves, in the form of step functions. The median absolute deviation is actually not sensitive to contaminant values above 1.1 seconds whereas the interquartile range has a constant sensitivity to contaminant values over 1.6. Since most of the outliers in the stride period data were well above the mean, this difference explains the considerably lower sensitivity of the median absolute deviation to outlier influence.

From this example, we appreciate that estimators of gait variable spread (i.e. variability) should be selected with prudence. The popular but non-robust variability measures of standard deviation and coefficient of variation both have 0 breakdown points [51], meaning that only a single extreme value is required to drive the estimators to infinity. Indeed, as seen in Figure 2, the presence of a small fraction of outliers can unduly inflate our estimates of gait variability. Outlier management [52], with methods such as outlier factors [53] or frequent itemsets [54], represents one possible strategy to reduce unwanted variability when using these non-robust estimators. Apart from the addition of a computational step, this strategy introduces the undesirable effects of outlier smearing and masking [55], which need to be carefully addressed.

In contrast, outliers need not be explicitly identified with robust estimation, hence circumventing the above complications and abbreviating computation. The interquartile range and median absolute deviation, have breakdown points of 0.25 and 0.5, respectively [51]. Practically, this means that these estimators will remain stable (bounded) until the proportion of outliers reaches 25% and 50% of the sample size, respectively. To circumvent explicit outlier detection and its associated issues altogether, and in the presence of noisy data, which often result from spatio-temporal recordings and parameterizations of kinematic and kinetic curves, robust estimators may thus be preferable in the summary of gait variables.

### Non-gaussian distributions

Even in the absence of outliers, univariate gait data may not adhere to a simple, unimodal gaussian distribution. In fact, distributions of gait measurements and derived parameters may be naturally skewed, leptokurtic or multimodal [56]. Neglecting these possibilities, we may summarize gait data with location and spread values which do not reflect the underlying data distribution.

#### Semi-parametric estimation

**Figure 4:**
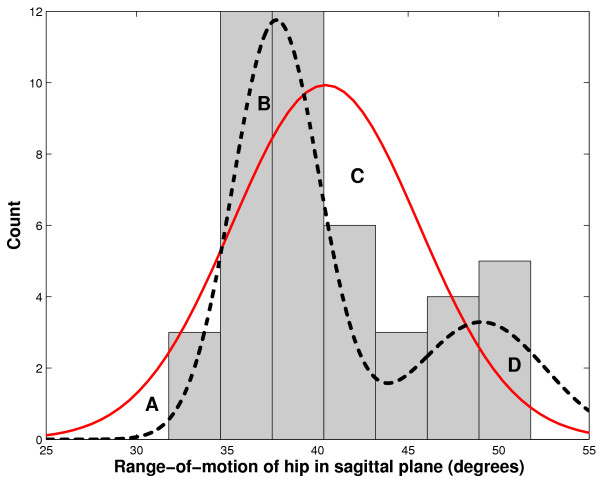
Multimodal parameter distribution. Shown here is a histogram of hip range-of-motion (45 strides from 9 able-bodied children) with two possible distribution functions overlaid: unimodal normal probability distribution (solid line) and bimodal gaussian mixture distribution (dashed line).

As an example, consider the hip range-of-motion extracted from 45 strides of 9 able-bodied children. A histogram of the data is plotted in Figure 4. Assuming that the data are gaussian distributed, we arrive at maximum likelihood estimates for the mean and standard deviation, i.e. 40.4 ± 5.1. However, the histogram clearly appears to be bimodal. A Lilliefors test [57] confirms significant departure from normality (*p *= 0.02). A number of approaches could be undertaken to find the underlying modes. One could perform simple clustering analysis [58], such as *k*-means clustering. Doing so reveals two well-defined clusters, the means and standard deviations of which are reported in Table 1. Alternatively, one could attempt to fit to the data, a convex mixture density of the form,

**Table 1:** Summary of bimodal ROM data

	Mixture distribution	*k*-means clustering	Normal distribution
Mode # 1	37.7 ± 2.4	37.7 ± 2.6	40.4 ± 5.1
Mode # 2	49.1 ± 3.5	47.7 ± 3.0	-
Mixing proportion (mode I/mode 2)	0.71/0.29	0.73/0.27	-
Critical value (lower)	33.35	32.96	30.40
Critical value (upper)	53.89	51.70	50.40



where *W*_*i *_is a scalar such that ∑_*i *_*W*_*i *_= 1 to preserve probability axioms, *N*_*C *_is the number of clusters or modes and  is a gaussian density with mean *μ*_*i *_and variance . The fitting of (6) is known as semi-parametric estimation as we do not assume a particular parametric form for the data distribution per se, but do assume that it can modeled by a mixture of gaussians. In the present case, *N*_*C *_= 2 and we can use a simple optimization approach to determine the parameters of the mixture. In particular, we determined the parameter vector [*W*_1_, *W*_2_, *μ*_1_, *σ*_1_, *μ*_2_, *σ*_2_] to minimize the objective function , where *n*_*j *_is the number of points within an interval of length Δ around *x*_*j *_and *N *is the number of points in the sample. The latter term in the objective function is a crude probability density estimate [59]. As seen in Table 1, the results of fitting this bimodal mixture yields similar results to those obtained from clustering.

What are the implications of naively summarizing these data with a unimodal normal distribution? First of all, the probabilities of observing range-of-motion values between 35 and 39 degrees, where most of the observations occur, would be underestimated. Likewise, ROM values between 39 and 48 degrees, where the data exhibit a dip in observed frequencies, would be grossly overestimated. These discrepancies are labeled as regions B and C in Figure 4. More importantly, the discrepancies in the tails of the distributions, regions A and D, suggest that statistical comparisons with other data, say pathological ROM, would likely yield inconsistent conclusions, depending on whether the mixture or simple distribution was assumed. Indeed, as seen in Table 1 the lower critical value of the simple normal distribution for a 5% significance level is too low. This could lead to exagerrated Type II errors. Similarly, the upper critical value is not high enough, potentially leading to many false positive (Type I) errors.

The above example depicts bimodal data. However, the mixture distribution method can be applied to arbitrary non-normal data distributions, regardless of the underlying modality. Fitting such distributions can be accomplished by the well-established expectation-maximization algorithm [60]. For a comprehensive review of other semi-parametric and non-parametric estimation methods, see for example [59].

#### Parametric estimation

**Figure 5:**
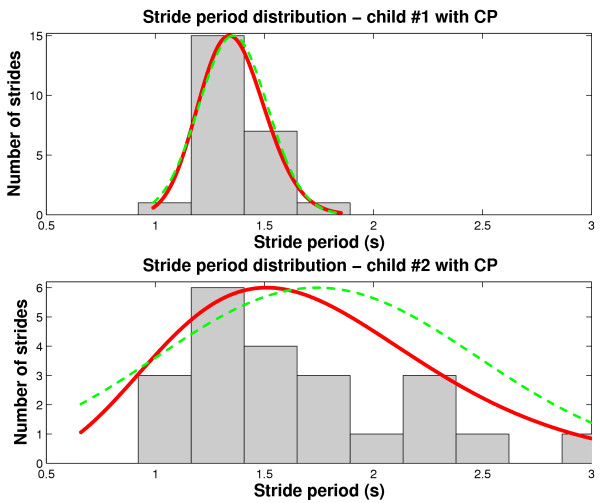
Comparison of stride period distributions between 2 children with spastic diplegia. In each graph, the dashed line is the normal probability distribution estimated for the data. The solid line is the gamma distribution fit to the data.



When we have some *a priori *knowledge about the underlying data distribution, we can adopt a simpler approach to summarize the gait data. In particular, we could fit the data to a specific parametric form. As an example, consider the task of comparing two sets of stride period data from two children with spastic diplegia, with identical gross motor function classification scores [61]. The histograms of strides for both children are shown in Figure 5. It is known that stride period data tend to be right-skewed [56]. A careful examination of the bottom graph indicates that the histogram is indeed right-skewed. In fact, the skewness value is 1.7 and Lilliefors test for normality [57] confirms significant departure from normality (*p *< 10^-5^). We thus determine the maximum likelihood gamma distribution for these data. The gamma distribution has the following parametric form [62],

where *a *is the shape parameter, *b *is the scale parameter and Γ(·) is the gamma function. The gamma distribution fits are plotted as solid lines in Figure 5.

As in the previous example, we consider the consequence of assuming that the data are normally distributed. Do these two children have similar stride periods? To answer this question, one may hastily apply a t-test, assuming that the stride period distributions are gaussian. The results of this test reveal no significant differences (*p *= 0.31), as reported in Table 2. To visualize the departure from normality, the maximum likelihood normal probability distribution fits to the stride data are superimposed on each histogram as a dashed curve. Note that the tails of the distribution are overly broad, particularly in the bottom graph. This diminishes the likelihood of detecting genuine significant differences between the data sets. Table 2 summarizes the maximum likelihood estimates of the distribution parameters under the two different distributional assumptions. Under the gamma distribution assumption, the stride periods between the two children are statistically different (*p *= 0.036) according to a Monte Carlo simulation of differences between 10^4 ^similarly distributed gamma random variables, which contradicts the previous conclusion. We have arbitrarily chosen the gamma distribution in this example as it appears to describe well the positively skewed data. However, there are many other parametric forms that could be fit to gait data in general. See for example [62,63].

**Table 2:** Statistical comparison of stride periods under different distributional assumptions

Child	No. strides	Gaussian distribution		Gamma distribution	
		
		*u*_*Z*_	*σ*_*Z*_	*a*	*b*
1	24	1.36	0.158	79.19	0.0171
2	23	1.74	0.734	7.513	0.232
		*p *= 0.31		*p *= 0.036	

In brief, the issue of non-normal distributions of measured gait variables or derived parameters, may lead to inaccurate reports of population means and variability and error-prone statistical testing. In fact, as the last example has shown, different distributional assumptions may lead to different statistical conclusions. Without a priori knowledge about the form of the distribution, one possible solution is to use a general mixture distribution to summarize the gait data. When we have some a priori knowledge about the underlying distribution, we can simply summarize the data using a known non-gaussian distribution, such as the gamma distribution exemplified above for the right-skewed stride period data. In either case, it is generally advisable to routinely check for significant departure from normality using such tests for normality as Pearson's Chi-square [64] or Lilliefors [57].

We remark that mixture models typically have a larger number of parameters than simple unimodal models. As a general rule-of-thumb, one should thus consider that mixture models generally require more data points for their estimation [59]. In particular, note that in any hypothesis test, the requisite sample size is dependent on the anticipated effect size, the desired level of significance and the specified level of statistical power [65]. For specific guidelines and methodology relating to sample size determination, the reader is referred to literature on sample size considerations in general hypothesis testing [66], normality testing [67], and other distributional testing [68].

## Single-cycle gait curves

Kinematic, kinetic and metabolic data are often presented in the form of single-cycle curves, representing a time-varying value over one complete gait cycle. Time is often normalized such that the data vary over percentages of the gait cycle rather than absolute time. Examples include curves for joint angles, moments and powers, ground reaction forces, and potential and kinetic energy. Due to variability from stride-to-stride, these measurements do not generate a single curve, but a family of curves, each one slightly different from the other. We will consider a family of gait curves as realizations of a random function [69-71]. Let *X*_*j *_(*t*) denote a discrete time function, i.e. a gait curve, where for convenience and without loss of generality, *t *is a positive integer and *t *= 1,..., 100. We further assume that the differences among curves at each point in time are independently normally distributed. Each sample curve, *X*_*j *_(*t*), can thus be represented as [70],

*X*_*j *_(*t*) = *f*(*t*) + *ε*_*j *_(*t*) *j *= 1,..., *N t *= 1,..., 100     (8)

where *f*(*t*) is the true underlying mean function, *ε*_*j *_(*t*) ~  (0, *σ*_*j *_(*t*)^2^) are independent, normally distributed, gaussian random variables with variance *σ*_*j *_(*t*)^2 ^and *N *is the number of curves observed. With this formulation in mind, we now address four prevalent challenges in analyzing gait curves, namely, undesired phase variation, robust estimation of spread, the difficulty with landmark analysis and lastly, the comparison of curves as whole objects rather than as disconnected points.

### Phase variation

It has been recognized that within a sample of single-cycle gait curves, there is both amplitude and phase variation [71-73]. Typically, when we describe variability in gait curves, we refer to amplitude variability. However, unchecked phase variation, that is the temporal misalignment of curves, can often lead to inflated amplitude variability estimates [72,73]. Computing cross-sectional averages over a family of malaligned gait curves can lead to the cancellation of critical shape characteristics and landmarks [74]. This issue presents a significant challenge when summarizing a series of curves for clinical interpretation and treatment planning. On the one hand, the presentation of a large number of different curves can be overwhelmingly difficult to assimilate. On the other hand, a prototypical average curve which does not reflect the features of the individual curves is equally uninformative.

Curve registration [71] is loosely the process of temporally aligning a set of curves. More precisely, it is the alignment of curves by minimizing discrepancies from an iteratively estimated sample mean or by allineating specific curve landmarks. Sadeghi et al. demonstrated the use of curve registration, particularly to reduce intersubject variability in angular displacement, moment and power curves [72,73]. Additionally, they reported that curve characteristics, namely, first and second derivatives and harmonic content were preserved while peak hip angular displacement and power increased upon registration [72]. This latter finding confirms that averaging unregistered curves may eliminate useful information.

Judging by the few gait papers employing curve registration, the method appears largely unknown among the quantitative gait analysis community. Here, we briefly outline the the global registration criterion method [71,75].

Since each gait curve is a discrete set of points, it is useful to estimate a smooth sample function for each observed sample curve. Given the periodic nature of gait curves, the Fourier transform provides an adequate functional representation of each curve. The basic principle is then to repeatedly align a set of sample functions to an iteratively estimated mean function. The agreement between a sample function and the mean function can be measured by a sum-of-squared error criterion. The goal of registration is to find a set of temporal shift functions such that the evaluation of each sample function at the transformed temporal values minimizes the sum-of-squared error criterion. The sample mean is re-estimated at each iteration with the current set of time-warped curves. As an optimization problem, the curve registration procedure is the iterative minimization of the sum-of-squared criterion *J*,



where *N *is the number of sample curves, *T *is the time interval of relevance, *w*_*i*_(·) is the time-warping function and  is the iteratively estimated mean based on the current time-warped curves *X*_*i *_(*w*_*i *_(*s*)). For greater methodological details, the reader is referred to [71,72,75]. This global registration criterion method is only one of several possibilities for curve alignment. Related methods which are applicable to gait data include dynamic time warping based on identified curve landmarks [41] and latency corrected ensemble averaging [28].

**Figure 6:**
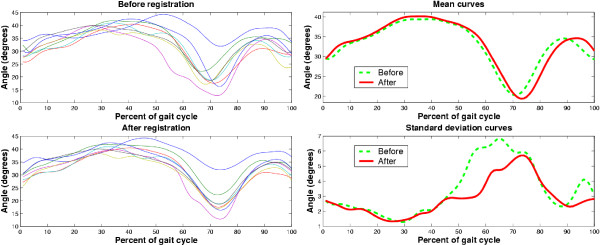
Accounting for phase variation. On the left, we portray unregistered (top graph) and registered (bottom graph) ankle angle curves from a child with spastic diplegia. On the right are the mean (top) and standard deviation (bottom) curves before (dashed line) and after (solid line) curve registration.

We exemplify the impact of accounting for undesirable phase variation using ankle angular displacement data from a child with spastic diplegla. The top left graph of Figure 6 depicts the unregistered curves, exhibiting excessive dorsiflexion throughout the gait cycle and the absence of the initial valley during loading response. Below this graph are the aligned curves. Note particularly the alignment of the large valley at pre-swing and the peak in swing phase.

The right column of Figure 6 indicates that the differences in the mean and standard deviation curves before and after registration are non-trivial, with maximum changes of +15% and -51%, respectively. The post-registration mean curve not only exhibits heightened but shifted peaks (3 – 5% of the gait cycle). This observation suggests that simple cross-sectional averaging without alignment may not only diminish useful curve features but can also inadvertently misrepresent the temporal position of key landmarks. Inaccurate identification of these landmarks, such as the minimum dorsiflexion at the onset of swing phase in this example, could be problematic when attempting to coordinate spatio-temporal and EMG recordings with kinematic curves. The bottom right graph shows a dramatic decrease in variability after registration, particularly in terminal stance. This finding is in line with the tendency towards variability reduction reported by Sadeghi et al. [72].

While curve registration is useful for mitigating unwanted phase variation in gait curves, there may be instances where phase variability is itself of interest [3]. In such instances, curve registration can still be useful in providing information about the relative temporal phase shifts among curves. Because curve registration actually changes the temporal location of data, it should not be applied in studies concerned with temporal stride dynamic characterizations, such as scaling exponents [21] or Lyapunov exponents [44]. At present, only a few gait studies have applied curve registration to manage undesired phase variability. However, the evidence in those studies, along with the example above, supports further research and exploratory application of curve registration to fully grasp its merits and limitations in quantitative gait data analyses. For now, curve registration appears to be the most viable solution to the challenge of summarizing a family of temporally misaligned gait curves. In the ensuing sections, we will demonstrate how curve registration can be used advantageously, in conjunction with other methods to address other curve summary and comparison challenges.

### Robustness of spread estimation

We have already seen that curve registration can mitigate amplitude variability in a family of gait curves. The robust measurement of variability in gait curves is itself a non-trivial challenge. One may need to estimate the variability in a group of curves for the purposes of classifying a new observation as belonging to the same population, or not [15]. Alternatively, knowledge of the variability among curves can help in the statistical comparison of two populations of curves [16], say arising from two different subject groups or pre- and post-intervention.

As in gait variables, the challenge lies in robustly estimating the spread of a sample of gait curves and to avoid fallacious under or overestimation. The intuitive and perhaps most popular way of estimating curve variability is the calculation of the standard deviation across the sample of curves, for each point in the gait cycle. This yields upper, *U*_*X*_, and lower bands, *L*_*X*_, around the sample of curves, i.e.

*U*_*X *_(*t*) = *μ*_*X *_(*t*) + *σ*_*X *_(*t*) *t *= 1,..., 100

*L*_*X *_(*t*) = *μ*_*X *_(*t*) - *σ*_*X *_(*t*)     (10)

where , for *t *= 1,..., 100, is the sample mean curve. Lenhoff et al. argued, by way of empirical examples and systematic cross-validation, that standard deviation bands provide inadequate coverage of the sample curves [15]. They instead supported the use of bootstrap prediction bands [76] which, in their study, provided close to the targeted 90% coverage of the sample curves. Two subsequent studies [16,17] have adopted the 90% bootstrap bands for the classification of new curves and for the comparison between groups of curves. The usefulness of bootstrap prediction bands for clinical identification of pathological deviations in kinematic curves has also been demonstrated [77]. In this section, we provide further evidence to support the use of bootstrap prediction bands and argue that they are more stable than standard deviation bands.

The basic idea of the bootstrap method is to create a large number of bootstrap subsets by resampling the curves *X*_*j*_, *j *= 1,..., *N *with replacement. For each subset, the bootstrap mean and standard deviation are calculated. One then checks how many of the sample curves are "covered" by the bootstrap standard deviation bands. A curve is considered covered, if its maximum absolute standardized difference from the bootstrap mean is less than the bootstrap constant *C*. The number of covered curves averaged over all the bootstrap subsets then yields the coverage probability for the given bootstrap constant, *C*. The upper and lower bootstrap prediction bands can then be written as,





The reader is referred to [15] for details for practical computer implementation of the above procedure.

**Figure 7:**
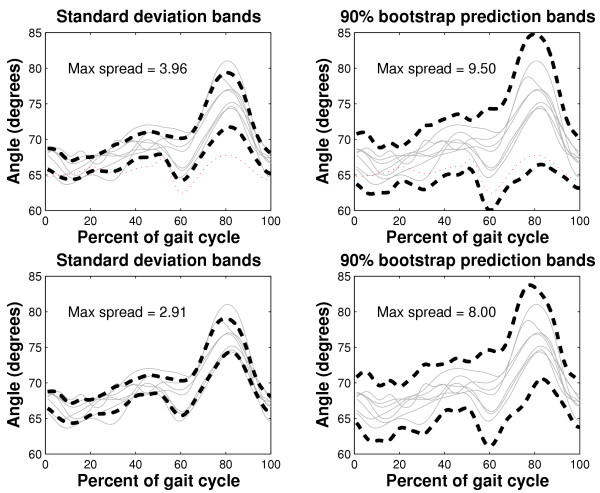
Estimation of spread in a group of registered knee angle curves from a 13-year old child with spastic diplegia. The left column depicts the standard deviation bands with (top graph) and without (bottom graph) an apparent outlying curve (thin dashed line). The 90% bootstrap prediction bands are plotted on the right, again with (top graph) and without (bottom graph) the outlying curve.

To exemplify issues of robust spread estimation, we consider knee angle curves from a child with spastic diplegia. Initially standard deviation and bootstrap bands are computed for the data prior to curve registration. The maximum absolute deviation from the sample mean curve is reported in Table 3. For both methods, the maximum spread decreases significantly upon registration, suggesting that there is significant inflated variability in the unaligned curve sample. Once the curves are aligned, one suspicious curve, plotted as a thin dashed line in Figure 7, becomes evident. The standard deviation bands around the sample with and without this outlying curve are shown on the left side of Figure 7. The maximum spread, that is max_*t *_ and *C*, for standard deviation and bootstrap bands, respectively, are labeled on each graph. We see that by removing the outlying curve, both the standard deviation and bootstrap bands become narrower. In fact, as seen in Table 3, the maximum standard deviation decreases by a dramatic 27%. Thus it appears that the variability among a group of curves, as estimated by both standard deviation and bootstrapping, can be minimized by curve registration and further reduced by the subsequent removal of outlying curves.

**Table 3:** Maximum spread estimates: registered and unregistered data

	Bootstrap bands		Standard deviation bands	
Data set	max *C*	Change	max	Change

unregistered data	12.5	-	4.7	-
registered data	9.5	-24%	3.96	-16%
registered data without outlier	8.0	-16%	2.91	-27%

**Figure 8:**
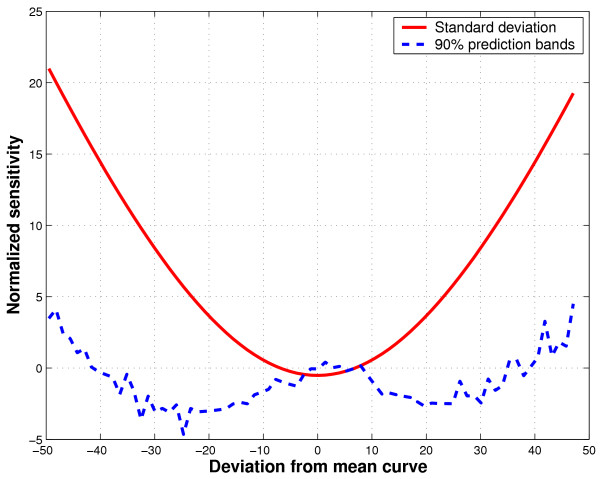
Sensitivity curves for the standard deviation bands and 90% bootstrap estimated prediction bands. Here, each point on a sensitivity curve represents the difference between the maxima of the bands estimated with clean and contaminated data.

To further understand the robustness properties of the two spread estimators, we generate sensitivity curves using the 45 knee angle curves introduced in Figure 4. These curves are first registered to minimize unwanted phase variability. In the case of gait curves, the contaminant is not a single point, but an entire curve. For convenience, we choose the following contaminant,



where *δ *∈ ℝ and *δ*_*min *_≤ *δ *≤ *δ*_*max*_. In other words, the contaminant is just a shifted version of the sample mean curve, . For simulating the sensitivity curve, we choose *δ*_*min *_= -50 and *δ*_*max *_= 50, recognizing that in practice, we would never observe deviations of this magnitude. This large range does however, gives us a more complete picture of the sensitivity curves. We proceed to define the sensitivity curves for the standard deviation and bootstrap estimates as follows,





where  is the variance of the uncontaminated sample and



is the variance of the contaminated sample. In the above,  is the mean curve of the contaminated sample. The notations *C*_*X *_and *C*_*X*, *z *_represent the bootstrap constants determined using the original and contaminated data, respectively. In other words, these sensitivity curves will reflect the influence of a contaminant curve, *z*(*t*), on the maximum estimated spread across a group of curves, over the gait cycle. Figure 8 summarizes the results of evaluating (15) over the simulated contaminants defined in (13).

We note that, as in the univariate case, the standard deviation exhibits quadratic sensitivity with vertex at the zero deviation curve. This parabolic sensitivity curve indicates that the standard deviation bands are not locally robust to contaminant curves. In contrast, the sensitivity curve for the bootstrap bands is not smooth and quartic in nature. The lack of smoothness is due to the random resampling inherent in the bootstrap method, such that with each contaminant curve, slightly different bootstrap samples are used in estimating the 90% prediction bands. Initially, as the contaminant curve deviates from the mean curve, the sensitivity is negative, meaning that the width of the estimated bands are smaller than those for the uncontaminated data. Indeed, the actual value of the bootstrap constant initially decreases, likely to counter the accompanying sharp increase in the standard deviation bands. In other words, as the standard deviation bands widen, a smaller bootstrap constant is required to cover 90% of the sample curves. However, as the contaminant curve deviates farther from the mean, the slope of standard deviation sensitivity increases in magnitude more slowly. With a smaller change in standard deviation band per unit of deviation of the contaminant curve, the bootstrap constant necessarily increases to maintain 90% coverage. This reasoning accounts for the subsequent increase in the tails of the bootstrap sensitivity curve. Finally, we note that overall, the bootstrap sensitivity curve, although apparently unbounded, traverses a much smaller range than the standard deviation curve. This would suggest that with the kinematic data employed in this example, the bootstrap coverage bands enjoy greater stability than their highly sensitive standard deviation cousins.

In brief, the foregoing discussion further supports the use of bootstrap coverage bands in robustly summarizing the variability within a family of gait curves. Moreover, curve registration and outlier removal can further tighten the location of the prediction bands.

### Problems with simple parameterizations

**Figure 9:**
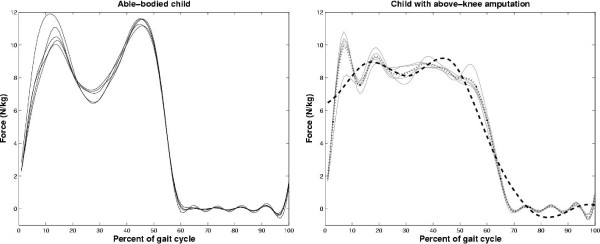
Inconsistency in multiplicity and location of local extrema. Graphs portray registered vertical ground reaction force curves from an able-bodied child (left) and from a child with above-knee prosthesis (right). The dotted line on the right is the mean curve while dashed line is the wavelet reconstructed mean curve.

It is common to compare specific landmarks or features of gait curves to gauge the impact of an intervention or to determine differences among different subject populations. However, the identification of curve features is inherently problematic. Indeed, the multiplicity of peaks and valleys across two different groups of curves may be inconsistent. As an example, Figure 9 portrays the vertical ground reaction force of an able-bodied child on the left with the typical loading response peak, mid-stance valley and terminal stance peak [78]. On the right is the vertical ground reaction force from the intact side of a child with an above-knee amputation, walking with a prosthetic lower limb. Note that there are at least three distinct peaks in the mean curve (thin dotted line). Attempts to compare the able-bodied and amputee force profiles are encumbered by the unclear choice of corresponding peaks. This is a common pitfall of comparing gait curves on the basis of specific landmarks.

The wavelet transform has been touted as a useful method for uncovering intrinsic trends in data [79,80]. Hence, it may be possible to extract an underlying low frequency trend from the amputee force curve for the sake of striking a comparison with the able-bodied curve. To this end, we decomposed the mean force curve for the child with amputation using a 4 – level coiflet wavelet transform [81]. We reconstructed the force curve using only the approximation coefficients. The resulting trend line is plotted on the right graph of Figure 9 as the thick dashed line and more closely resembles the expected force profile. Extraction of the extrema yields plausible peak locations at 17% and 44% of the gait cycle and a valley at 30%. These locations are comparable to those for the able-bodied child (peaks at 12% and 44% and valley at 26% of the gait cycle), but suggest a slightly extended loading response phase.

The extraction of the trend line in this example illustrates that in some curves, the desired landmarks may be concealed by the fluctuations of higher frequency signal components and hence may be salvageable. However, even when landmarks are clearly identifiable among curves, they reflect only a very microscopic view of the entire curve. For example, two curves could have identical landmarks, but pronounced differences in shape characteristics. We therefore do not advocate the isolated use of simple parameterizations or landmarks for routine comparison of curves. Rather, the comparison of two sets of curves should be based on the entire curve and not isolated parameterizations. We suggest however, that landmark analysis and simple parameterizations can be meaningful as a post-hoc procedure, i.e. investigating how curves are similar or different, only *after *statistically significant differences among curves or lack thereof have been established. We therefore suggest to first statistically compare entire gait curves as unified objects, and reserve landmarks for post-hoc analysis. In the following section, we describe how such a statistical test may be carried out.

### Comparison of gait curves as coherent entities

If gait curves were strictly deterministic, one could simply define a distance measure between two curves and be done. However, due to stride-to-stride variability, an extension of the univariate statistical test is needed, to determine if one set of curves could have arisen from the same statistical distribution as another. Alternatively, one could test whether the average difference between two sets of curves is approximately zero, within the critical values of an expected distribution of differences. The fundamental challenge is to compare families of gait curves as coherent entities rather than as unconnected, independent points. One way to consider curves as a whole rather than as disjoint points is to give them an appropriate functional representation. One can then compare the functional representations of the curves. Exploiting this principle, Fan and Lin [70] proposed a general method for comparing two sets of discrete time-sampled curves. In their method, the discrete Fourier transform of the standardized difference between the mean curves of two families of curves is computed. Only selected low frequency components of the transform, which encompass the majority of signal energy, are retained. These coefficients are then subjected to the adaptive Neyman test which yields the probability that the two families of curves have similar means. To the best of our knowledge, the adaptive Neyman statistic [69] has not yet been applied in the gait literature for the comparison of empirical gait curves. We therefore outline below, in some detail, the proposed procedure that we have adapted from Fan and Lin [70]. Suppose that we would like to compare two families of gait curves, {*X*_*j *_(*t*), *j *= 1,..., *N*_*X*_} and {*Y*_*j *_(*t*), *j *= 1,..., *N*_*Y*_}, with *t *= 1,..., 100. The null hypothesis is that the difference between the means of the two families of curves is zero. In the random function formulation given by Equation (8), we can write, *H*_0 _: *f*_*X *_(*t*) - *f*_*Y *_(*t*) = 0, where *f*_*X *_(*t*) and *f*_*Y *_(*t*) are the true underlying mean curves. For notational convenience, we will let *t *= 0,..., *T *- 1, where we have chosen *T *= 100 in the previous examples. The main steps of the test are as follows.

1. Compute the sample mean curves, *μ*_*X *_(*t*) and *μ*_*Y *_(*t*), where  and likewise for *μ*_*Y *_(*t*)

2. Compute the sample variance curves,  and , where  and likewise for .

3. Align the two mean curves using the global registration criterion method. Denote the registered curves as  and . This step does not appear in the original formulation of Fan and Lin [70].

4. Compute the standardized difference *Z*(*t*) between the registered means,



5. Compute the discrete Fourier decomposition, , of the standardized difference,





where *k *= 0,..., *T*/2, Real(·) and Imag(·) denote the real and imaginary components of the complex Fourier coefficient , respectively, and *k *denotes the Fourier frequency.

6. Form a new vector of coefficients **E**, of length *T *+ 1, by pairing real and imaginary coefficients of the complex Fourier coefficients, , as follows,



7. Estimate the adaptive Neyman statistic, *T*_*AN*_(**E**) for the vector defined above. This proceeds in two steps.

(a) Determine the optimal the number of coefficients to retain to maximize , where *E*_*i *_are the elements of the vector defined above and 1 <*m *<*T *+ 1. This optimal value of *m*, denoted , maximizes the power of the adaptive Neyman statistic [70]. The maximum statistic value is written as,



where *Var*(*E*^2^), is the variance of the square of the elements of *E *obtained in step 6.

(b) Let *K *= ln(*T *ln *T*). Compute the following final transformed test statistic value [70],



Here, we have explicited indicated that the statistic has been computed for the vector **E **of Fourier coefficients. Asymptotically, this statistic has an exponential of an exponential distribution [69], that is, *P*(**T**_*AN *_≤ *x*) → exp(-exp(-*x*)), as *T *becomes arbitrarily large.

8. Estimate the p-value of the computed test statistic value, *T*_*AN*_(**E**), by Monte Carlo simulation of a large number, say 10^6^, of vectors, **Y**_*i*_, *i *= 1,..., 10^6^, each of length *T *and whose elements are drawn from a standard normal distribution, i.e. **Y**_*i *_~ (0, 1), ∀*i*. The rationale is that when two sets of curves arise from the same random function, the standardized differences of their Fourier coefficients are normally distributed around 0. For each normal vector, **Y**_*i*_, evaluate *T*_*AN*_(**Y**_*i*_) as in step 7 above. When the null hypothesis of no differences is true, the probability of observing an adpative neyman statistic as extreme as *T*_*AN*_(**E**) is estimated as,



where *H*(·) is the heaviside function, where *H*(*x*) = 1 only if *x *> 0 and is 0 otherwise. In the examples below, we simulated 10^6 ^such vectors to estimate the probability of observing *T*_*AN*_.

**Figure 10:**
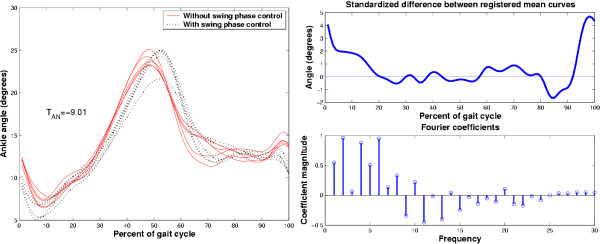
Comparison of ankle angle curves (left pane) from an above-knee amputee using a prosthetic knee without (solid line) and with (dashed line) a swing phase control mechanism. The right pane depicts the standardized difference between the registered mean curves for the two groups (top graph) and the corresponding first 30 coefficients of its discrete Fourier transform.

We exemplify the above procedure with two kinematic data sets taken from a child with an above-knee amputation, wearing two different types of prosthetic devices. Of interest is whether the use of a swing phase control mechanism within the prosthetic knee affects gait. Figure 10 depicts the ankle angular displacements with (dashed line) and without (solid line) the swing phase control mechanisms. By visual inspection, the curves look similar in magnitude and exhibit a slight phase difference. We would expect statistical testing to conclude that the curves are not different. The top right graph is the standardized difference between the registered mean curves. Note that most values fluctuate around 0. The bottom right graph is a stem plot of selected Fourier coefficients of the Fourier transform of the standardized difference. Note the concentration of energy in the low frequencies and the distribution of coefficients above and below 0. It is thus not surprising that the result of the adaptive Neyman test statistic, yielded *T*_*AN *_= -9.01 which corresponds to *p *= 1 for  = 2. In other words, it is very likely that the two groups of curves came from the same distribution of random functions. In the present context, the swing phase mechanism had no effect on the ankle angular displacement.

**Figure 11:**
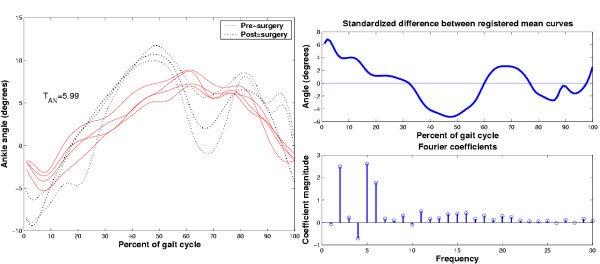
Comparison of ankle angle curves (left pane) from one individual before and after total ankle replacement surgery. The right pane portrays the standardized difference between the registered mean curves of each group (top graph) and the first 30 coefficients of its discrete Fourier transform.

To illustrate the statistical detection of differences, we draw upon a second example involving kinematic curves from an adult subject pre- and post-surgical replacement of the ankle. In Figure 11, the pre-surgery curves do not have well resolved peaks or valleys whereas post-surgically, distinct peaks and valleys emerge with substantial magnitude. On the basis of this visual inspection, one would anticipate that statistical testing should indicate that the pre- and post-surgery curves are indeed different. The standardized difference between the registered mean curves exhibits relatively large fluctuations around 0 and the retained Fourier coefficients are nearly all positive, resulting in a positively skewed coefficient distribution. The adaptive Neyman statistic value for these coefficients is *T*_*AN *_= 5.99 corresponding to *p *= 2.6 × 10^-5 ^with  = 6. Hence, the statistical test indicates that there is strong evidence for rejecting the null hypothesis. It appears that surgery has significantly altered the gait curves. Once significant statistical difference has been established, one can then seek to identify specific characteristics which differentiate the two sets of curves. For example, the post-surgical curves exhibit a well-defined valley, towards plantar flexion at toe-off and a strong first dorsiflexion peak in terminal stance. Both of these extrema are absent in the presurgery curves.

Note that we have not said anything about the requisite sample sizes for the statistical comparison of gait curves. Clearly, as in unidimensional power analysis [65], the required sample size depends on the effect size, significance level and specified power. To the best of our knowledge, no power-sample size tables have been derived for the adaptive Neyman statistic at the time of writing. For insights on the topic, the interested reader can refer to authoritative works [65,82] on power analysis in the univariate case. The statistical testing demonstrated here can be extended to compare more than two groups of curves, using high-dimensional analysis of variance [70]. Further, when the standardized difference curve is not smooth, wavelet denoising can be used to identify the frequency bands where the majority of signal energy is concentrated [69]. The adaptive Neyman statistic introduced here is only one of several possibilities for objectively and rigorously testing differences among curves. Other alternatives include an ANOVA test for functional data [83] and functional canonical correlation analysis [84]. The procedure outlined in this section formalizes the comparison of gait curves as coherent entities. The method provides a means of statistically confirming overall similarities and differences that we may detect by visual inspection, but may have difficulty quantifying with conventional time and frequency domain parameterizations.

## Recommendations

**Figure 12:**
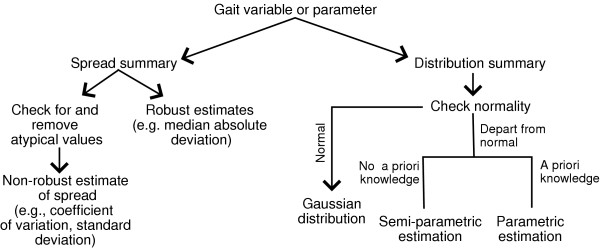
Heuristic guidelines for summary of gait variables or parameters.

We summarize the foregoing discussions by proposing some heuristic guidelines for dealing with the aforementioned variability issues in gait variables and curves. For gait variables or parameters, the suggested solution pathways are shown in Figure 12.

**Figure 13:**
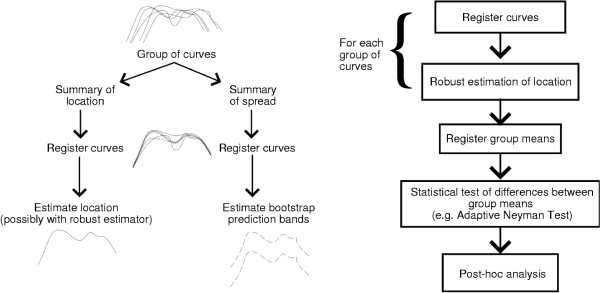
Heuristic guidelines for the summary (on the left) and comparison (on the right) of gait curves.

For gait curves, the suggested procedures for summary and comparison are summarized in Figure 13. A few comments beyond the above discussions are in order. Note that robust estimation is suggested in the summary of gait curves, as after registration, there may still be curves which appear atypical, in amplitude or overall shape. Location estimation of gait curves was only discussed in the context of the adaptive Neyman test, but is included in Figure 13 for completeness. In the comparison of curves, post-hoc analysis would encompass the comparisons of conventional curve parameterizations or landmarks (e.g. peaks and valleys), as investigative procedures to explain the formally established statistical differences or lack thereof.

## Future directions

This paper has only skimmed the tip of the iceberg in the discussion and demonstration of several promising analytical approaches for practically addressing variability issues in gait data summary and comparison. The topics of curve registration and bootstrap estimates of curve variability, although not necessarily new to gait data analyses, have been seldom studied and applied in the gait research community. The handful of studies to date on these subjects, have provided strong initial evidence for potentially improving the rigor and objectivity of gait data interpretation. Examples in the present paper lend further credence to these methods. Systematic comparisons of these techniques with conventional parameterizations, summary statistics, and even expert interpretation of gait data, would lead to a greater appreciation of their relative merits and limitations in gait data analyses. For example, would the use of registration and bootstrapping to consolidate gait data improve the consistency of clinical decision-making? Given the propensity for variability inflation in gait data, the topic of robust estimation needs to be studied in greater depth, in terms of contaminant influences and possibly adaptive estimators [49]. Likewise, the rigorous statistical comparison of gait curves as coherent entities rather than uncorrelated sets of points, is a promising area of research in gait variability analyses. This stream of study is only in the embryonic stages but promises to strengthen the comparison of quantitative gait data and to complement its subjective interpretation, a pratice which has been debated in literature [85-87].

## Authors' contributions

T. Chau wrote the entire manuscript and carried out the majority variability analyses reported herein.

S. Redekop collected most of the empirical data reported herein, carried out all the kinematic and kinetic data analyses, wrote programs for data extraction, identified the datasets and helped in their interpretation.

S. Young provided the literature review for the manuscript and contributed significantly to revising various drafts.
